# Commentary: M-Autonomy

**DOI:** 10.3389/fpsyg.2018.00680

**Published:** 2018-05-30

**Authors:** Krzysztof Dołȩga

**Affiliations:** Institut für Philosophie II, Ruhr University Bochum, Bochum, Germany

**Keywords:** mental autonomy, misrepresentation, predictive procesing, philosophy, self-model

## Introduction

In his recent article, Metzinger (2015) argues for a new construal of mental or M-autonomy as a functional property which consists in the deployment of a special kind of model, one which represents the self as an epistemic agent. This epistemic self-model is “[…] a global model of the cognitive system as an entity that *actively* constructs, sustains, and controls knowledge relations to the world and itself.” (Metzinger, 2015, p. 272, all emphasis original unless stated otherwise).

The aim of this commentary is to evaluate Metzinger's proposal from the perspective of the recently popular predictive processing (or PP) framework (Friston, 2010; Clark, 2013, 2016; Hohwy, 2013). Such analysis is needed because the implications of PP for mental autonomy and agency are still unclear, despite the framework's growing popularity. In his work, Metzinger alludes to the framework on several occasions (Metzinger, 2015, 2017), but such references are not comprehensive enough to reveal the full consequences that adoption of PP would have for his position. Although both views share the basic assumption that cognition consists predominantly in construction and deployment of mental models, other aspects of the framework, such as the nature of action under active inference (Wiese, 2017), may carry surprising consequences for Metzinger's proposal.

## M-autonomy

On Metzinger's picture, the fundamental aspect of M-autonomy is that it allows for mental agency. As Metzinger points out: “[s]ome mental activities are not autonomously controllable, because one centrally important defining characteristic does not hold: they cannot be inhibited, suspended, or terminated.” (Metzinger, 2015, p. 275). Thus, M-autonomy differs from unintentional mental behavior by allowing for voluntary control of occurrent thoughts and mental processes. Metzinger calls this feature “veto control” or “intentional inhibition” and stresses that “[…] it is a functional property which we do not ascribe to the brain, but to the person as a whole.” (Metzinger, 2015, p. 278). It is the loss of precisely this control, when we “zone out,” daydream, or undergo non-lucid dreaming, which leads him to conclude that “[…] a recurring *loss* of mental autonomy is one major characteristic of our cognitive phenomenology.” (Metzinger, 2015, p. 276).

Although the ability to inhibit mental behaviors is a personal-level phenomenon, it is a direct consequence of the meta-representational capacities of the cognitive system. Metzinger considers mental agency as involving a control component, which he refers to as “a second-order mental action” (Metzinger, 2015, p. 277). He elaborates on this in the following way:

“The satisfaction conditions of second-order mental actions are constituted by successfully influencing other mental actions or mental behaviors, first-order mental processes are the targets of second-order mental action. Examples of second-order mental action are the termination of an ongoing violent fantasy, but also the deliberate strengthening and sustaining of a spontaneously arising pleasant daydream, the effortful attempt to make an ongoing process of visual perception more precise by selectively controlling the focus of attention […].” (Metzinger, 2015, p. 277).

Mental agency, therefore, is a second-order representational faculty which takes first-order mental processes as its objects. As the above passage implies, such second-order mental representations have content (i.e., satisfaction conditions) which is satisfied when the desired outcome regarding certain lower-order mental state(s) or process(es) is achieved^1^. In case of veto control, such representation is satisfied when (depending on its exact content) the target mental process is either suspended or terminated.

Metzinger's construal of autonomy is also representational in another sense. As has been suggested in the introduction, it involves a special kind of representation in which the subject represents itself as an epistemic agent engaged in constructing and searching “[…] for new epistemic relations to the world and [themselves]” (Metzinger, 2015, p. 280). This kind of self-representation is referred to as an “epistemic agent model” or EAM and is crucial for sustaining the first-person perspective of an active agent (Metzinger, 2015, p. 281)^2^. What makes the EAM relevant for M-autonomy and agency is that the subject gains ownership of first-order mental processes because such processes are embedded into this special kind of self-model:

“If these processes are additionally represented as control processes, as successful acts of exerting causal influence, they can now be consciously experienced as processes of self-control or instances of successful *self*-determination. An EAM is an instrument in what one might call ‘epistemic auto-regulation’: it helps a self-conscious system in selecting and determining what it will know, and what it will not know.” (Metzinger, 2015, p. 282).

The EAM is central to Metzinger's position regarding the loss of M-autonomy, which he calls a ‘collapse of the EAM’. He stresses that, although one way of describing such situations would be to treat them as cases in which the subject has completely lost the ability to control their own thoughts, it is not the position he endorses (Metzinger, 2015, p. 282). His view is that the loss of M-autonomy consists in a loss of a specific “form of knowledge” rather than the capacity for second-order control of mental behavior (Metzinger, 2015, p. 282). The potential for mental agency is still present during wayward episodes, but what the subject lacks is a kind of “explicit and globally available *representation* of an existing functional ability for active epistemic self-control […]” (Metzinger, 2015, p. 282). In other words, the subject lacks the self-awareness of their ability to control first-order processes, something that can be only acquired through an EAM.

One interesting consequence of this characterization of mental agency and its absence is that it becomes difficult to explain how subjects can regain mental autonomy. If the subjects does not know they possess the ability to control their occurrent mental processes, then how can they regain such knowledge and, more importantly, how can they act on it? The slightly paradoxical explanation provided by Metzinger is that the reemergence of an EAM and the subsequent regaining of M-autonomy must be caused by an unconscious mental process or event (Metzinger, 2015, p. 283). This explanation can be finessed by pointing out that usual cases of mind-wandering do not involve a complete loss of a first-person perspective. This implies that most episodes of absent autonomy involve only a partial collapse of the EAM, one in which the “*implicit* knowledge about the relevant potential” remains intact (Metzinger, 2015, p. 283). Metzinger claims that one usually does not notice the absence of M-autonomy, “[b]ecause we confuse our abstract, retrospective, and purely intellectual knowledge that, in principle, we had the critical mental ability all along with what actually was the case on the level of concrete, inner phenomenology: the absence of an EAM.” (Metzinger, 2015, p. 282–283).

I will return to this and previous points in section Mental Actions as Misrepresentations, but for now I would like to stress that, on Metzinger's view, regaining M-autonomy is a kind of cognitive illusion in which the subject fails to apprehend a “blink” in their self-representation and erroneously assumes that they were able to deploy effective veto control all along^3^.

## Attention and action in PP

By now, the PP framework has been firmly established in the literature on philosophy of mind and cognitive science (Clark, 2013, 2016; Hohwy, 2013; see also Wiese and Metzinger, 2017 for a helpful introduction). Due to space restrictions, I will focus only on the details necessary for the argument in the subsequent sections.

The main idea behind PP is that cognition consists in a hierarchically organized process of generating predictions about the states of the sensory periphery and minimizing the error of said predictions (Rao and Ballard, 1999). Unlike in more traditional cognitive architectures, predictions are deployed in a top-down manner, influencing and constraining the activity on lower levels, while the only information propagated upwards is the error signal encoding the difference between the actual and expected states. Importantly, such prediction error signals need to be weighed according to their expected precision (here understood as the inverse of estimated variance) since different environmental conditions and sensory modalities can vary with regard to the levels of noise and uncertainty present in the sensory stimulus. On PP, this process of optimization of precision estimates has been used for modeling attention (Friston and Stephan, 2007; Feldman and Friston, 2010; see also Hohwy, 2012 for a helpful and detailed discussion). A PP system can minimize prediction error in two different ways. In *passive inference* the system revises and updates the generative model of the world from which erroneous predictions have been generated in order to issue more accurate ones. However, as Hohwy notes: “[…] a system without agency cannot minimize surprise but only optimize its models of the world” (Hohwy, 2012, p. 3). This is why the system needs to also perform *active inference*, which involves bringing the states of the sensory periphery closer to the expectations of the current generative model^4^. This can be done by manipulating the body and acting in the world in order to bring the predicted sensory state about. Under active inference predictions are treated as sensorimotor representations of the intended consequences of action (by encoding the states that the sensory receptors are expected to be in), allowing the system to endogenously initiate behavior in a way similar to ideomotor or common coding theories of motor control (see e.g., Wolpert and Flanagan, 2001 or Hommel et al., 2001, respectively).

Active inference, however, presents a serious problem for the framework. This issue has been explored by Wanja Wiese who pointed out that:

“[S]ensorimotor estimates […] corresponding to intended (but not yet performed) movements are in conflict with perceptual input. When I am intending to move my arm, the brain will predict the sensory effects this movement will have. […] These predicted signals are in conflict with the sensory signals the brain is actually receiving […]. The result is a prediction error.” (Wiese, 2017, p. 1240).

As Wiese notes, a PP system can have two ways of resolving the influx of error signals caused by such counterfactual motor predictions. One is to simply perform passive inference and revise the expectations in a way which will accommodate the error. This, however, would not result in any bodily movement. To carry out the intended action, the system needs to ignore the error signals so that the expected state of the sensory periphery can be brought about. In Wiese's words:

“The essential aspect of this account is that peripheral sensory precision estimates (that function as weights for prediction errors) must be turned town during action (otherwise action is inhibited). This, however, means that the precisions of peripheral sensory signals are systematically *underestimated* prior to movement onset […].” (Wiese, 2017, p. 1241).

In other words, in order to move it is necessary to tune down the sensory evidence that one is not moving. The required attenuation of sensory precision provides a formal description of sensory attenuation; namely, the reduction of the perceived intensity of stimuli caused by one's own action. Crucially, subscribing to an attenuation of precision as the explanation for sensory attenuation in psychophysics, reveals a fundamental link between sensory attenuation and sensory attention. This follows from the fact that sensory attention is thought to be mediated by increases in sensory precision that rest upon top-down predictions of precision; i.e., the second-order statistics that underwrite the confidence placed in ascending sensory prediction errors (Feldman and Friston, 2010; Brown et al., 2013).

The inverse relationship between initiating action and the estimated precision of bottom-up input can be demonstrated on the example of the force matching task (Shergill et al., 2003; cf. Wiese, 2017). In this experimental paradigm subjects are supposed to match an externally applied force by either using their hands or a remote-controlled device. The results of the experiment show that subjects systematically underestimate the self-generated force they apply in the direct condition, a result which is anticipated by the PP account of sensory feedback attenuation during action production.

The intimate link between attention and action has the surprising consequence of casting *both* peripheral sensory precision estimates *and* the sensorimotor expectations initiating actions as systematic misrepresentations. Although such misrepresentations are beneficial for the system (in the sense of McKay and Dennett, 2009; see Wiese, 2017 for more details), the implication that, in order to act, a PP system needs to misrepresent its own states has significance for Metzinger's position on M-autonomy and its dependence on mental action.

## Mental actions as misrepresentations

To see how Wiese's point about the nature of action bears on the notion of M-autonomy in PP it is best to start with the investigation of the place that the EAM occupies in the cognitive economy postulated by the framework. This issue has been explicitly addressed by Metzinger, who proposed that:

“[…] what the cognitive self-model continuously *predicts* (Friston, 2010; Hohwy, 2013; Clark, 2016) are just much more abstract aspects of reality, in a wider temporal frame of reference, and not ongoing events on the sensory sheet. The [self-model] can be seen as an integrated global hypothesis about the state of the system in which it appears, constituted by a large number of individual predictions or sub-hypotheses, which are hierarchically structured and optimized at different timescales. A conscious self-model is therefore composed of different layers of expectations, in a continuous attempt of minimizing uncertainty and prediction error related to the system itself.” (Metzinger, 2015, p. 287, references adapted)

As this passage implies; the self-model is constituted or composed of the parts of the predictive hierarchy which are removed from the sensory periphery. At first glance, such a characterization seems to bar the worry that the EAM will involve the same kind of systematic misrepresentation as the PP account of motor control. After all, the sensorimotor misrepresentations which enable motor control must employ peripheral sensory precision estimates, which do not figure in the construal of the EAM provided by Metzinger. Recall, however, that the EAM is also supposed to be a control model which allows the subject to exert influence on other mental processes in a way similar to that in which sensorimotor control models allow the subject to exert influence on the body and the world. As has been said in previous section, the self-model allows for mental agency because it represents lower-order mental processes as controllable by the subject. This means that mental agency must share the crucial aspect of the PP conception of action—it must involve the generation of predictions about lower-order processes in order to influence their behavior. Such control predictions, including predictions of sensory precision, involve counterfactual contents (as well as context).

This feature of mental action can be brought into focus once we look at what the EAM is supposed to represent. On PP, the meta-cognitive nature of EAM means it consists of expectations or hypotheses which target (i.e., take as their intentional object) other states in the system. However, in order to manipulate or exercise “veto-control,” the higher-order model must represent the desired outcome of such lower-order mental states. The conditions of satisfaction for the higher-order representations guiding mental action are fulfilled once the first-order states and processes are the way they are expected to be, i.e., once the top-down predictions from the higher levels of the hierarchy constrain their activity. Thus, the self-model must initially misrepresent these states, presenting them as being in a (counterfactual) state the system is expected to be in.

The similarity with bodily action applies also to the issue of down-regulating the expected precision of prediction errors. In the case of bodily movement, such regulation is necessary in order to “[…] attend away from the evidence that we are not moving [and] to enable our predictions to be fulfilled.” (Wiese, 2017, p. 1240). If we consider that this top-down control is mediated by the attenuation of the (expected) precision of prediction errors throughout the predictive processing hierarchy, we arrive at a nice metaphor for mental action; namely, selecting which ascending prediction errors to attend to or attenuate. On this picture, higher order processes exercise control by attenuating the errors produced by certain target (lower-order) processes to free high-level processing from precise lower level constraints. The attenuation of bottom-up signals is needed so that the higher-order control model can represent and issue predictions about the lower-order state(s) or process(es) as being in the “intended” state. Crucially, such a PP formulation of mental agency (and M-autonomy) involves the higher levels of the system misrepresenting the content of lower-order ones, at least when mental actions are initiated.

To illustrate this point, let us assume that I am switching the focus of my attention from task or thought *a* to task or thought *b*. For this mental action to occur, the higher-order EAM must misrepresent me as already attending to *b* and it must also attenuate the evidence that I am, in fact, currently attending to *a*. As this simple example shows, endogenous attentional control involves explicitly misrepresenting the current (lower-order) mental process in order to influence it. Putting this in the broader context of Bayesian inference, the kind of misrepresentation involved can be interpreted as a posterior belief that differs from a belief that is informed by un-attenuated, precise prediction errors (i.e., sensory evidence). This misrepresentation underlies our ability to act on the world; namely, by fulfilling (mis)representations of what we believe we should be doing. On this view, the same misrepresentational capacity emerges under mental action which attenuates the precision of prediction errors at the intermediate levels of hierarchical processing^5^.

Although this is a surprising consequence, one which has not been anticipated by Metzinger, it may not be completely unwelcome for his view. Recall, that subjects are unaware of regaining M-autonomy due to a “self-representational blink” which accompanies the collapse of the EAM. In other words, they misrepresent themselves as having had the capacity for effective mental action all along. This somewhat counterintuitive implication of Metzinger's view fits well into the PP interpretation of M-autonomy, since in order to control mental processes, via the optimization of (second order) predictions, the EAM must misrepresent the (first-order) self. In this context, it is much less surprising that regaining this meta-cognitive capacity should include the system misrepresenting itself. Such retrospective misrepresentation may be a result of the PP system striving to increase the coherence of the self-model while minimizing its complexity over time.

I would like to end this section with a short note about the empirical tractability of the implications of a PP interpretation of M-autonomy. As has been mentioned in section 2, the PP account of action coheres well with the results of the force matching task illusion. Could there be a similar effect for mental action as well? A stipulative answer is that the misrepresentation driven nature of mental action may be the source of some of the systematic biases involved in self-assessment and introspective judgement. One example of such influence could be the reluctance to adjust one's estimates of an uncertain variable when one is confident in their ability to make a precise judgement (Mannes and Moore, 2013). In such cases the lowered meta-cognitive awareness of one's own uncertainty may stem from the fact that the higher-order representation of one's ability involved in making and sustaining a judgement depends on the attenuation of lower-order error signals which would otherwise help to adjust the uncertainty of the judgement. Importantly, a similar overconfidence effect has also been shown in an anticipatory motor control task (Mamassian, 2008), implying that the lack of sensitivity of one owns uncertainty is not confined to judgements alone. The PP interpretation of M-autonomy may also have consequences for how we understand attention deficit disorders. It is possible that in cases such as ADHD the subjects' ability to effectively attenuate the bottom-up signals is impaired, limiting the control that the self-model can exert on lower-order cognitive and perceptual processes.

## Conclusion

Analyzing Metzinger's account of M-autonomy from the perspective of the PP framework reveals that the capacity for initiating mental actions involves misrepresenting one's own mental states. Indeed, Metzinger seems to acknowledge this issue by explicitly talking about “the self-deception model of goal selection and action initiation” (Metzinger, 2017, p. 19). Nevertheless, while the capacity for such misrepresentation may be beneficial to the PP system in the long run (as in the case discussed by Wiese, 2017), it casts doubt on Metzinger's proposal that M-autonomy relies on a veridical self-model of epistemic import.

One of the benefits of a PP interpretation of Metzinger's view is that it helps us make sense of his postulate that we are largely unaware of the episodes of lost autonomy, and that regaining it involves a retrospective illusion which presents one as having been M-autonomous all along. However, this positive outcome of applying PP to Metzinger's view may come at a high price, putting his wider claim about M-autonomy being a result of self-modelling the subject as an epistemic agent into question. If exercising M-autonomy is also triggered by a process of misrepresenting one's own mental states, then M-autonomy might itself be an illusion—indeed, on this view, all perception of endogenously generated actions may be illusory to some extent.

## Author contributions

The author confirms being the sole contributor of this work and approved it for publication.

### Conflict of interest statement

The author declares that the research was conducted in the absence of any commercial or financial relationships that could be construed as a potential conflict of interest.

## Footnotes

^1^I will use the terms “state” and “process” interchangeably, but this is not meant to signify a metaphysical difference.

^2^The concept of the EAM is firmly anchored in Metzinger's prior work on phenomenal self-models (Metzinger, 2003, 2007, 2008). Space constraints preclude me from discussing the commitments of this view in detail.

^3^Please note that there is a tension in Metzinger's account of how autonomy is regained. The loss of M-autonomy is a loss of a kind of personal knowledge about one's abilities, not the abilities themselves. However, regaining autonomy results in a retrospective judgement that the subject had the ability all along. This judgement is simultaneously correct in one sense and wrong in another. It is correct (on this account) that the subject had the ability all along, but it is not true that they could have acted on it.

^4^As Wiese correctly notes (Wiese, 2017, p. 1239) the overall mathematical framework of active inference is meant to formally subsume what has been referred to as passive inference.

^5^Metzinger (2017) and Wiese (2017) make the defensive argument that, due to their direction of fit, representations of the intended goals of actions should not be counted as misrepresentations from a diachronic perspective. However, the same defense may not be available in the case of the second-order statistics of expected precision, where the system must simply ignore the expectation about the bottom-up signal.
